# Prognostic significance of serum chemerin levels in patients with non-small cell lung cancer

**DOI:** 10.18632/oncotarget.14956

**Published:** 2017-02-01

**Authors:** Chun-Hua Xu, Yang Yang, Yu-Chao Wang, Jun Yan, Li-Hua Qian

**Affiliations:** ^1^ Endoscopic Center of Nanjing Chest Hospital, Nanjing, Jiangsu 210029, China; ^2^ Clinical Center of Nanjing Respiratory Diseases and Imaging, Nanjing, Jiangsu 210029, China; ^3^ Department of Respiratory Medicine, Affiliated Jiangning Hospital of Nanjing Medical University, Nanjing, Jiangsu 211100, China; ^4^ MOE Key Laboratory, Model Animal Research Center, Nanjing University, Nanjing, Jiangsu 210061, China; ^5^ Department of Respiratory Medicine, Nanjing Pukou Central Hospital, Nanjing, Jiangsu 211800, China

**Keywords:** chemerin, non-small cell lung cancer, prognosis, diagnosis, biomarker

## Abstract

Chemerin plays an important role in adipogenesis and chemotaxis of the innate immune system. The aim of this study was to explore the significance and prognostic value of serum chemerin levels in patients with non-small cell lung cancer (NSCLC). Serum specimens from 189 NSCLC patients and 120 healthy controls were collected. The levels of serum chemerin were measured by sandwich enzyme-linked immunosorbent assay (ELISA). The serum chemerin levels were significantly elevated in NSCLC patients compared with healthy controls (*P* < 0.001). Higher serum chemerin levels were associated with advanced TNM stage, lymph node metastasis, and distant metastasis. Area under receiver operating characteristic curve (ROC) for serum chemerin was 0.809 (95% CI: 0.722–0.896) at a sensitivity of 0.624 and of specificity 0.675. The cut-off value of chemerin was 1500 pg/ml for discriminating NSCLC from healthy controls. Kaplan-Meier log rank analysis revealed that the higher serum chemerin patients had a shorter overall survival (OS) and progression-free survival (PFS) compared with lower chemerin patients (*P* = 0.004, *P* = 0.001, respectively). Further univariate and multivariate Cox regression analysis showed that serum chemerin was an independent risk factor of prognosis of NSCLC patients. In conclusion, measurement of chemerin might be a useful diagnostic and prognostic biomarker for NSCLC patients.

## INTRODUCTION

Lung cancer is one of the most common cancers worldwide and has the first leading cancer-related mortality with much poorer survival [1]. Non-small cell lung cancer (NSCLC) constitutes approximately 80% of total lung malignancies. Despite significant advances in multidisciplinary treatment modes, the 5-year survival rate of lung cancer is less than 15% [2, 3]. So far, only a few prognostic factors, such as sex, performance status, disease stage, and weight loss, have been identified [4]. Currently, using clinical parameters alone, we cannot accurately predict the clinical outcome of lung cancer patients. The discovery of molecular biological prognostic factors may aid in a more accurate prediction of clinical outcome of patients with NSCLC.

Chemerin is a novel adipokine, which plays an important role in adipogenesis and chemotaxis of the innate immune system [5]. Previous studies have indicated that chemerin expressions are downregulated in many carcinomas and are associated with poor differentiation [6–9]. However, other studies demonstrate that the expressions of chemerin are upregulated in grade III/IV glioma tissues and overexpression of chemerin in orals quamous cell carcinoma is correlated with poor clinical outcomes of patients [10, 11]. Recent research has shown that the levels of plasma chemerin in patients with lung cancer were significantly higher than those in healthy control [12]. However, the prognostic significance of chemerin expression in blood specimens from NSCLC patients has not yet been determined.

In this study, we investigated the correlation between serum chemerin levels and clinicopathological characteristics and survival. Our results showed that the evaluation of serum chemerin could be a valuable diagnostic and prognostic biomarker for NSCLC.

## RESULTS

### Clinical characteristics of patients

The mean age of the NSCLC patients (61.8 years) was not obviously different from healthy controls (62.6 years). The proportion of male gender accounted for 65.6% of the NSCLC patients and 57.5% of the healthy controls, respectively, with no significantly difference. At the time of diagnosis, 79 patients were at stage I + II and 110 patients were at stage III + IV, 53.4% of the patients with lymph node metastases, and 31.7% with distant metastases. Patient's data were analyzed after a 5-year follow-up, and the median OS was 30 months, and median DFS was 21 months. The patient’ s characteristics are presented in Table 1.

**Table 1:** Clinicopathological variables of NSCLC patients and healthy controls

Variables	NSCLC patients (*n* = 189)	Healthy control (*n* = 120)
Age (years)	61.8 ± 11.2	62.6 ± 8.9
Gender (*n*, %)		
Male	124 (65.6)	69 (57.5)
Female	65 (34.4)	51 (42.5)
Histology		
ADC	107(56.6)	
SCC	82 (43.4)	
TNM stage		
I + II	79 (41.8)	
III + IV	110 (58.2)	
Differentiation		
Well-moderate	120 (63.5)	
Poor	69 (36.5)	
Lymph node metastases		
Negative	88 (46.6)	
Positive	101(53.4)	
Distant metastases		
Negative	129 (68.3)	
Positive	60 (31.7)	

Abbreviations: ADC, Adenocarcinoma; SCC, Squamous cell carcinoma.

### Association between serum chemerin levels and clinicopathological variables

**Figure 1:**
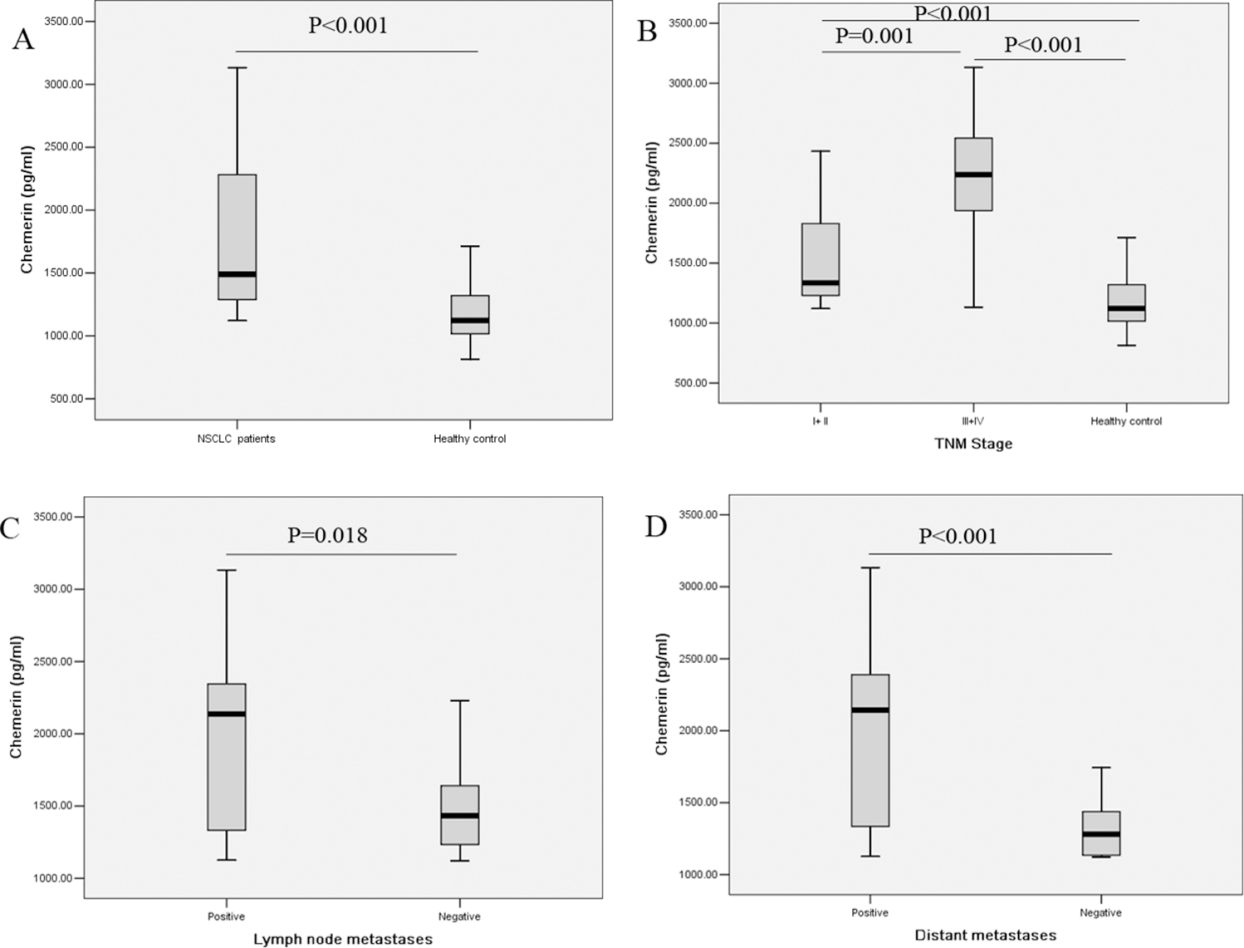
Comparison of serum chemerin levels (**A**) between healthy controls and NSCLC patients; (**B**) in healthy controls and NSCLC patients at different TNM stage; (**C**) in NSCLC patients with and without lymph node metastasis; (**D**) in NSCLC patients with and without distant metastasis.

As shown in Figure 1A, the levels of serum chemerin were significantly higher in NSCLC patients compared with healthy controls (1783.16 ± 568.06 pg/ml vs. 1195.08 ± 229.94 pg/ml, *P* < 0.001). We further evaluated the clinicopathological significance of the serum chemerin levels in NSCLC patients. The association between the serum chemerin levels and clinicopathological variables in NSCLC patients were summarized in Table 2. As displayed in Figure 1B, when compared with the healthy controls, the serum chemerin levels were elevated in NSCLC patients at both early stage and advanced stage, and serum chemerin levels were even higher in advanced stage patients than those of early stage patients. Furthermore, the serum chemerin levels were obviously higher in patients with lymph node metastases than those without (Figure 1C, Table 2). Meanwhile, statistically significant differences in chemerin levels were found between NSCLC patients with distant metastases and those patients without distant metastases (Figure 1D, Table 2). Besides, the serum chemerin levels have no significant differences from other clinicopathological variables (Table 2). After all, these results indicated that serum chemerin levels increased in NSCLC patients, and associated with the progression and metastasis NSCLC, which could be serve as a potential biomarker to differentiate NSCLC patients from healthy controls, even the indicator for prognosis.

**Table 2:** Association between serum chemerin levels and characteristical variables in NSCLC patients

Factors	Number	Chemerin (pg/ml)	*P*- value
Age (years)			0.406
≥ 60	102	1872.21 ± 591.28	
< 60	87	1717.35 ± 554.20	
Gender			0.537
Male	124	1729.55 ± 542.69	
Female	65	1842.42 ± 604.05	
Histology			0.246
ADC	107	1660.59 ± 481.93	
SCC	82	1873.76 ± 618.83	
Differentiation			0.193
Well-moderate	120	1874.48 ± 587.40	
Poor	69	1630.96 ± 517.44	
TNM stage			0.001*
I + II	79	1529.54 ± 434.70	
III + IV	110	2163.59 ± 540.34	
Lymph node metastases			0.018*
Negative	88	1514.49 ± 380.02	
Positive	101	1944.36 ± 606.60	
Distant metastases			0.000*
Negative	129	1424.67 ± 332.11	
Positive	60	2179.39 ± 510.86	

*Statistically significant difference (*P* < 0.05).

Abbreviations: ADC, Adenocarcinoma; SCC, Squamous cell carcinoma.

### Diagnostic value of serum chemerin levels in NSCLC patients

**Figure 2:**
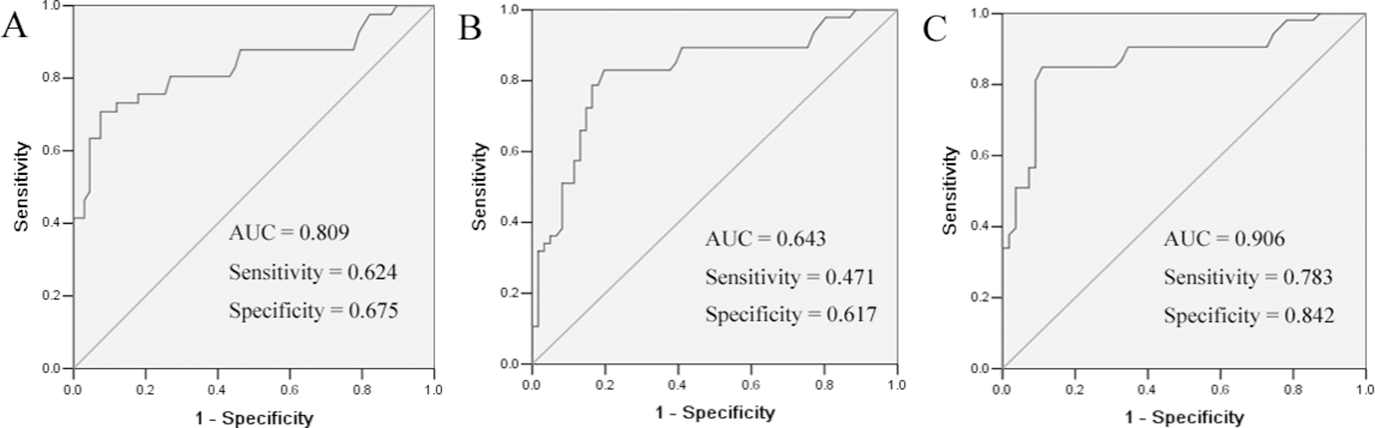
ROC curves for the serum chemerin (**A**) and CEA (**B**) and chemerin + CEA (**C**) in differentiating NSCLC patients and healthy controls. The areas under the curve of serum chemerin, CEA and chemerin + CEA were 0.809, 0.64 and 0.906, respectively.

To assess the performance of serum chemerin as a marker, ROC curves were used to calculate the sensitivity of this marker in separating NSCLC patients from healthy controls. As shown in Figure 2A, an area under the curve (AUC) value for serum chemerin reached 0.809 (confidence interval (95% CI) 0.722–0.896). With a cut-off value of 1500 pg/ml, serum chemerin has a sensitivity of 62.4%, a specificity of 67.5%. These results indicated that serum chemerin was a valuable biomarker for NSCLC diagnosis.

The diagnostic threshold afforded by the ROC analysis for CEA was 5.0 ng/ml. The area under the CEA ROC was 0.643 (95% CI 0.644–0.841). It was lower compared with the areas of chemerin (Figure 2B). The combined diagnostic value of chemerin and CEA in NSCLC was further detected. The results showed that the combined detection of these two indices had a sensitivity of 78.3% and a specificity of 84.2%. The combination of chemerin and CEA produced better sensitivity and specificity than chemerin and CEA alone (Figure 2C).

### Serum chemerin levels are an independent prognostic indicator for NSCLC patient survival

**Figure 3:**
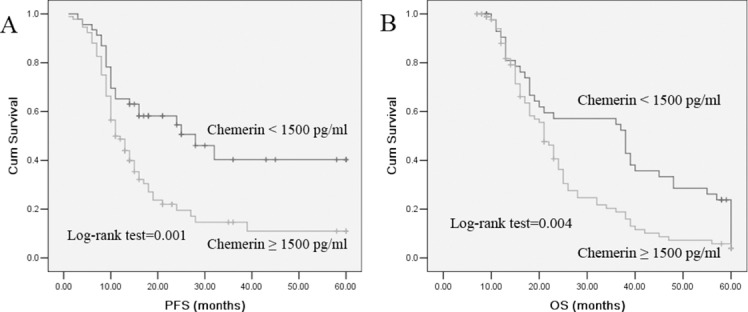
Kaplan–Meier survival curves for PFS and OS in patients with chemerin -high and -low NSCLC Log-rank test determined that the PFS and OS in high chemerin group were significantly longer than those in the low chemerin group (*P* = 0.001; *P* = 0.004).

To evaluate the prognostic significance of the serum chemerin levels, we used serum chemerin cut-off value 1500 pg/ml, which was calculated from previous ROC analysis, as a threshold to partitioned 189 NSCLC patients into two groups, high serum chemerin group (chemerin ≥ 1500 pg/ml, *n* = 118) and low serum chemerin group (chemerin < 1500 pg/ml, *n* = 71). Univariate analysis showed that serum chemerin levels were significantly correlated OS and PFS (Table 3). In multivariate analysis, high chemerin was found to be significantly associated with a shorter PFS and OS. Kaplan-Meier survival curves further demonstrate that lung cancer patients with high chemerin have substantially shorter PFS and OS, compared to those with low chemerin patients (Figure 3). As expected, TNM stage, lymph node metastases and distant metastases were found to be strongly associated with decreased PFS and OS, in both univariate and multivariate analyses.

**Table 3:** Univariate and multivariate Cox analysis of variables considered for PFS and OS of NSCLC patients

Variables	PFS			OS		
	HR	95% CI	*P*-value	HR	95% CI	*P*-value
**Univariate analysis**						
Age (≥ 60 vs. < 60)	1.061	0.803–1.404	0.676	1.144	0.900–1.453	0.271
Gender(male vs. famale)	1.187	0.884–1.594	0.255	1.222	0.529–2.870	0.645
Histology (ADC vs. SCC)	0.689	0.351–1.353	0.279	0.721	0.385–1.347	0.305
Differentiation (well-moderate vs. poor)	1.333	0.645–2.756	0.438	1.134	0.483–2.665	0.773
TNM stage (I + II vs. III + IV)	1.292	1.186–1.407	0.001*	1.700	1.345–2.147	0.001*
Lymph node metastases (positive vs. negative)	1.732	1.076–2.788	0.024*	1.943	1.099–3.435	0.022*
Distant metastases (positive vs. negative)	1.710	1.101–2.654	0.017*	1.815	0.907–3.633	0.024*
Chemerin (high vs. low)	1.859	1.494–2.314	0.001*	2.931	1.765–4.869	0.001*
**Multivariate analysis**						
Age (≥ 60 vs. < 60)	1.240	0.486–4.995	0.583	1.152	0.698–1.902	0.579
Gender (male vs. famale)	1.468	0.677–2.358	0.290	1.428	0.987–2.549	0.078
Histology (ADC vs. SCC)	0.671	0.346–1.320	0.238	1.186	0.734–1.916	0.487
Differentiation (well-moderatevs. poor)	1.016	0.603–1.712	0.952	1.848	0.519–4.247	0.462
TNM stage (I + II vs. III + IV)	1.600	1.129–2.691	0.008*	1.850	1.301–2.630	0.001*
Lymph node metastases (positive vs. negative)	2.133	1.153–3.944	0.016*	1.611	1.036–2.506	0.034*
Distant metastases (positive vs. negative)	2.623	1.264–4.085	0.001*	2.881	1.460–5.687	0.002*
Chemerin (high vs. low)	1.898	1.112–3.239	0.019*	2.339	1.377–3.974	0.002*

*Statistically significant difference (*P* < 0.05).

Abbreviations: PFS, Progression-free survival; OS, Overall survival; ADC, Adenocarcinoma; SCC, Squamous cell carcinoma; HR, Hazard ratio; CI, Confidence interval.

## DISCUSSION

In the present study, the diagnostic and prognosis value of serum chemerin levels in NSCLC patients were evaluated. We found that serum chemerin levels were obviously elevated in NSCLC patients compared with the controls. Unfavorable clinicopathological variables, TNM stage, lymph node metastasis, and distant metastasis were associated with high serum chemerin levels. Kaplan-Meier and Cox regression analysis revealed that high chemerin levels were correlated with poor survival and it could become an independent prognostic factor for NSCLC. Furthermore, ROC analysis displayed that serum chemerin had a potential to distinguish NSCLC patients from healthy controls. The combined diagnostic value of the chemerin and CEA in NSCLC was also analyzed. The results showed that the combined detection of these two indices had a better diagnostic value than the use of a single index. Our results suggest that serum chemerin may serve as a useful serum biomarker for the diagnosis and prognosis of NSCLC patients.

Adipokines are multifunctional peptide hormones that perform essential regulatory functions related to energy balance, satiety, and immunity [13, 14]. Many studies have demonstrated that adipokines play important roles in tumor angiogenesis, invasion, differentiation, and progression [15–17]. Chemerin, a member of adipokines, plays an important role in regulating angiogenesis, cell proliferation and migration, inflammation, glucose, insulin signaling, and lipid metabolism [18–20]. Although the dysregulated expression of chemerin in the tumor tissues has been reported, the results are inconsistent [6–11, 21]. One possible explanation for these inconsistencies is that chemerin expression may vary in different types of cancers, which are common phenomena in tumor biology [22].

So far, a large number of studies demonstrate the relationship between expression of chemerin in tumor tissues and the clinical outcome of some cancers [6–11]. However, there is a paucity of literature on the association of serum chemerin levels with prognosis of lung cancers. In the current study, serum chemerin levels were determined and found to be the independent prognostic factor for patients with lung cancer. Thus, the results of our study indicated that chemerin levels in blood might help for predicting the prognosis of patients with lung cancer.

In summary, our results showed that serum chemerin levels significantly elevated in NSCLC patients compared with the controls, and there was a strong association between high serum chemerin levels and TNM stages, lymph node metastasis, and distant metastasis. Furthermore, NSCLC patients with higher serum chemerin levels had poorer prognosis, suggesting that serum chemerin may be a useful clinical biomarkers in diagnosing, progression and prognostic evaluation in NSCLC.

## MATERIALS AND METHODS

### Patients

One hundred and eighty-nine NSCLC patients diagnosed and treated in the Nanjing Chest Hospital, from January 2010 to January 2014 were enrolled in this study. Patients who suffered hyperlipidemia, metabolic syndrome, infection, cardiovascular disease, liver disease, previous malignancy, or received adjuvant therapy before surgery were excluded from the study. All patients*’* histopathological classification was determined according to the WHO criteria, and staged classification was defined according to the 7th edition of UICC TNM staging system [23]. One hundred and twenty healthy controls were recruited from healthy unrelated subjects whose age and gender matched subjects who did not have any family history cancer were recruited in this study.

Follow-up lasted through December 2015, with a median follow-up period of 22 months for living patients (range, 3–60 months). Progression-free survival (PFS) was defined as the time interval between the date of diagnosis and the date of disease relapse. Overall survival (OS) was defined as the time interval between the date of diagnosis and the date of death.

The study protocol was approved by the ethics committee of Nanjing Chest Hospital. All patients provided written informed consent before enrollment.

### Measurement of serum chemerin and CEA levels

Serum samples from each individual were obtained at the time of diagnosis, before any therapeutic measures were started. Samples were centrifuged at 1500 × g for 20 min at −4°C. The supernatant was stored at −70°C for assessment of the levels of chemerin. The chemerin levels were determined by sandwich ELISA with the commercial chemerin ELISA kit (Mil-lipore, USA). The CEA levels were measured by electrochemiluminescence immunoassays. The normal upper limit for this assay was 5 ng/ml. All samples were blinded to the technologists running the assays, and the code was broken to the statisticians after the database was constructed.

### Statistical analysis

Statistical software (SPSS for Windows, version 18) was used for the analysis. All variables under normal distribution were shown as the mean ± standard deviation. The differences between groups were determined by Mann-Whitney *U* test, and Pearson chi-square test or Fisher's exact test was tested for categorical values. To determine the diagnostic accuracy of chemerin, receiver operating characteristic (ROC) curves was retrieved from logistic regression analysis and the area under the curve (AUC) was calculated. Univariate survival analysis was performed using the Kaplan-Meier method and the log-rank test. Multivariate analysis was conducted to determine an independent impact on survival using the Cox proportional hazard method. *P* < 0.05 was considered statistically significant.
